# Ten-Year Outcome of a Randomized Trial: Cytoreduction and HIPEC with Mitomycin C Versus Oxaliplatin for Appendiceal Neoplasm with Peritoneal Dissemination

**DOI:** 10.1245/s10434-024-16441-z

**Published:** 2024-11-12

**Authors:** Edward A. Levine, Heidy Cos, Konstantinos I. Votanopoulos, Perry Shen, Greg Russell, Paul Mansfield, Keith Fournier, David Bartlett, John H. Stewart

**Affiliations:** 1 Section of Surgical Oncology, Department of General Surgery, Wake Forest Baptist Health, Winston-Salem, NC USA; 2 Department of Biostatistics and Data Science, Wake Forest Baptist Health, Winston-Salem, NC USA; 3 M.D. Anderson Cancer Center, Houston, TX USA; 4 University of Pittsburgh, Pittsburgh, PA USA

**Keywords:** Appendiceal cancer, Pseudomyxoma, Chemotherapy, Oxaliplatin, Mitomycin C, HIPEC

## Abstract

**Background:**

Appendiceal cancer is a rare disease that has proven difficult to study in prospectively. Our initial report of this trial showed minor hematologic toxicity with both mitomycin C and oxaliplatin and similar 3-year survival. We now report an update of the first prospective randomized trial for appendiceal cancer with 10-year follow up.

**Patients and Methods:**

Patients with mucinous appendiceal neoplasms and evidence of peritoneal dissemination were enrolled in the Multicenter Randomized Trial to evaluating HIPEC for 120 min with oxaliplatin (200 mg/M^2^) or mitomycin C (40 mg). Overall survival and disease-free survival were calculated at 10 years and compared between the groups.

**Results:**

A total of 121 patients were included in the study. The patients were 57% female, with a mean age of 55.3 years (range 22–82 years). The disease was low grade in 71% and high grade in 29%. The average peritoneal cancer index (PCI) score was 18 (SD 10) in the mitomycin C group and 17.9 (SD 9.4) in the oxaliplatin group (*p* = 0.94). The 10-year survival rate was 56.2% (SE 7.2) with mitomycin C and 47.5% (SE 8.4) with oxaliplatin, *p* = 0.83. The 10-year progression-free survival rate in the mitomycin C group was 45.2% (SE 8.4) compared with 50.4% (SE 6.7) in the oxaliplatin group, *p* = 0.95. Median survival was 9.1 years after HIPEC with oxaliplatin, and median not reached for the mitomycin C group (> 5.6 years).

**Conclusions:**

Oxaliplatin and mitomycin C have similar long-term efficacy for hyperthermic intraperitoneal chemotherapy (HIPEC) in patients with appendiceal neoplasms and peritoneal dissemination. Long-term survival is experienced by most patients after cytoreduction surgery (CRS) and HIPEC for appendiceal neoplasms.

Appendiceal mucinous neoplasms are rare, and without established screening systems, frequently present with peritoneal dissemination.^1–5^ There is great variability in the observed outcomes based on histologic type, tumor grade, and disease volume, with the best survival benefit observed in peritoneal surface disease (PSD) from low-grade appendiceal (LGA) primary lesions. However, even within the LGA group, there is significant variability, thus all appendiceal cancer is not equal.^6–9^

Cytoreduction surgery (CRS) and hyperthermic intraperitoneal chemotherapy (HIPEC) have become the mainstay of treatment for appendiceal mucinous neoplasms with peritoneal dissemination.^4,10,11^ Different chemotherapeutic agents have been used in the delivery of HIPEC. The two most common, oxaliplatin and mitomycin C, have been extensively studied.^11–15^ We have previously performed a phase 1 trial of oxaliplatin for HIPEC and found the maximally tolerated dose in a 120-min perfusion to be 200 mg/M^2^.^12^ Retrospective analyses have reached differing conclusions on which agent is superior.^13–16^

We performed and published the initial results from the first multicenter randomized trial comparing the use of standard dose of mitomycin C (40 mg) to a dose of oxaliplatin (200 mg/M^2^) compatible with a 2-h perfusion time as defined in a phase I trial.^6,11^ The initial report of this study found that oxaliplatin and mitomycin C have different hematologic toxicities. Mitomycin C was significantly associated with higher rates of leukopenia and oxaliplatin was significantly associated with higher rates of thrombocytopenia. Oxaliplatin was also reported to have better short-term quality of life by patients. However, both agents had similar short term overall and progression-free survival, and the complication profile was similar as well. We concluded that patients with baseline thrombocytopenia would be better treated with mitomycin C, while in those with preoperative leukopenia, oxaliplatin would be preferred.

In this study, we report the long-term survival of patients with appendiceal mucinous neoplasm undergoing cytoreduction and HIPEC randomized to oxaliplatin or mitomycin C who were included in the trial.

## Patients and Methods

Institutional Review Board approval was obtained. The trial was registered with the NCT (no. 1580410) and investigational drug approval was obtained for oxaliplatin (NCI-2009-00947). The methodology for the trial is described in our prior manuscript.^11^ However, briefly: the inclusion criteria for the trial were the following: biopsy proven appendiceal neoplasm with peritoneal metastases, recovery from any neoadjuvant systemic chemotherapy, resectable or resected primary, debulkable peritoneal disease, no extra-abdominal disease, Eastern Cooperative Oncology Group (ECOG) performance status ≤ 2, no prior CRS, and adequate hepatic, marrow and renal function. The exclusion criteria comprised: neuroendocrine carcinoma, peripheral neuropathy, prior radiation or investigational therapy, human immunodeficiency virus (HIV), hepatitis, or tuberculosis infections.

Data analysis included demographics, age, race, gender, ECOG performance status, R status of resection, type of malignancy, histologic grade, nodal status, comorbidities, preoperative or postoperative chemotherapy, volume of peritoneal disease, morbidity, mortality, and survival. Appendiceal primaries were grouped in cohorts on the basis of histologic grade (low or high)^17^ and were further subclassified on the basis of lymph nodal status. The presence of peripheral liver metastases, if readily resectable, was not considered a contraindication. All patients had a complete history and physical exam, tumor markers, and computed tomography (CT) of the chest, abdomen, and pelvis before CRS/HIPEC procedures.

The CRS/HIPEC procedure was conducted as previously described by our group using the closed abdominal technique.^18,19^ Briefly, HIPEC was preceded by a cytoreduction with a goal of resection of all gross tumor. A generous midline incision was utilized for all explorations. Resection of the peritoneum (where needed) was performed by stripping it off the abdominal wall or intraperitoneal organs as needed, combined with multivisceral resections (such as splenectomy, large and small bowel resection, hysterectomy, etc.) to effectualize maximal tumor debulking as determined by intraoperative findings.

Patients were cooled to a core temperature of about 34–35 °C by passive measures (i.e., not warming airway gases or intravenous solutions) during cytoreduction. After the completion of cytoreductive surgery, peritoneal perfusion catheters were placed as per institutional standard practice. Probes were utilized to monitor perfusate and core temperatures. The abdominal skin incision was temporarily closed with a running suture to prevent leakage of peritoneal perfusate. A perfusion circuit was established (typically with 3 L of crystalloid solution). The goal flow rate was at least 1 L/min. The outflow catheters were drained into a reservoir containing a coarse filter for debris and to reduce foaming. The heated chemotherapy solution was added and circulated through the institution’s standard perfusion equipment. The abdomen was gently massaged throughout the perfusion to improve drug distribution to all peritoneal surfaces.

In subjects randomized to the oxaliplatin arm, the agent was added to the perfusate once outflow temperatures exceed 39 °C at a dose of 200 mg/M^2^. Subjects randomized to the mitomycin C arm received 30 mg of mitomycin C once outflow temperatures exceed 39 °C. An additional 10 mg of mitomycin C was added to the perfusate 60 min into the HIPEC. The maximum inflow temperature of 42.5 °C was tolerated during the perfusion, with the target outflow temperature being 40 °C. The total perfusion time for both agents was 120 min. Following the perfusion, the peritoneum was washed out with 3 L of perfusate and the peritoneum passively drained. The skin was then reopened, and the cannulas removed under direct vision. The abdomen was then definitively closed after completions of required anastomoses or stomas.

After surgery, patients were followed with daily complete blood counts during their admission and at outpatient follow-up on postoperative day 30. Patients with white blood cell (WBC) < 4.0 were given G-CSF at 5 µg/kg daily until the WBC exceeded 10 k/mm^3^. Transfusions were delivered at surgeon’s discretion. Surgical morbidity and mortality were recorded according to the CTCAE 3.0 classification^20^ and Clavien–Dindo^21^ systems. R0 and R1 resections were grouped together as complete cytoreductions. Cytoreductions with residual macroscopic disease were characterized as R2 and subdivided on the basis of the size of residual disease (R2a ≤ 5 mm, R2b ≤ 2 cm, R2c > 2 cm).^22^

Participants were randomized to receive either 200 mg/M^2^ of oxaliplatin or 40 mg of mitomycin C via HIPEC. Samples of normal and malignant peritoneum were collected pre- and post-treatment for analysis. Participants were followed for toxicity starting 24 h after the end of surgery to 30 days after surgery, and for progression and survival after surgery. Patients were followed at least every 6 months with CT or magnetic resonance imaging (MRI), blood counts, functions, and a carcinoembryonic antigen (CEA) level through year 5 and annually thereafter up to year 10.

Descriptive statistics, including means and standard deviations for continuous data and frequencies and percentages for categorical data, were calculated for all study measures. Fisher’s exact tests were used to assess differences between groups in categorical variables, while independent Student’s *t*-tests were used to analyze continuous variables.

Overall survival (OS) was calculated from the date of CRS/HIPEC (or first CRS/HIPEC in cases where a patient underwent more than one procedure) to the last known date of follow-up or the date of death. The Kaplan–Meier method was used to provide survival estimates. The log-rank test of the chi-squared approximation was used to assess statistical significance. A *p*-value < 0.05 was assumed to be statistically significant. All analyses were performed using SAS 9.4 (Cary, NC, USA).

## Results

**Fig. 1:**
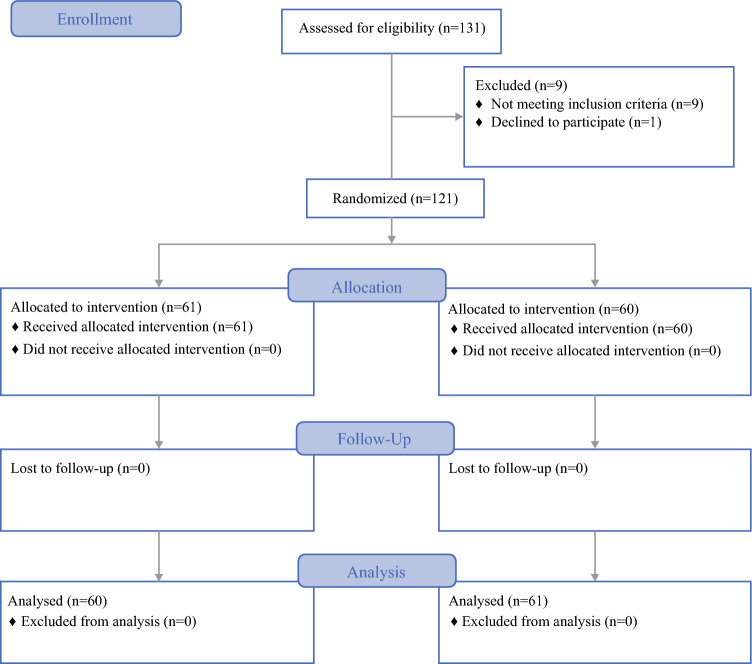
CONSORT diagram

This is a survival update on the population described in our multicenter randomized trial and treated at Wake Forest University. For practicality purposes, we will describe our population demographics again in this paper. The consort diagram is shown in Fig. 1.

A total of 121 eligible patients were recruited to the trial between 6 July 2009 and 15 October 2015. An additional nine patients consented, but were not evaluable, due to disease that could not be adequately debulked, thereby precluding HIPEC. Demographics are presented in Table 1. Of the evaluable patients, 57% were female and 43% male. The average age at diagnosis was 54.1 ± 13.0 years with a range of 21.9–81.7. The average age at the time of surgery was 55.3 ±  13.3 years with a range of 22.0–81.8 years (Table 2).

**Table 1:** Cohort characteristics

Characteristic	Mitomycin C (*N* = 61)	Oxaliplatin (*N* = 60)	*p*-value
Follow-up (median)	6.7 years	8.0 years	0.027
Sex, female,* n* (%)	36 (59)	33 (55)	0.72
Race, *n* (%)			
White	55 (90)	56 (93)	0.72
African American	5 (8)	3 (5)	
Asian	1 (2)	0	
Hispanic	0	1 (2)	
Age, years, mean (SD)	54.6 (14.0)	55.2 (12.5)	0.83
Peritoneal Carcinomatosis Index, mean (SD)	18.0 (10.0)	17.9 (9.4)	0.94
Resection status, *n* (%)			
R0	13 (21)	14 (23)	
R1	20 (33)	23 (38)	0.35
R2a	24 (39)	15 (25)	
R2b	4 (7)	6 (10)	
R2c	0	2 (3)	
Site,* n* (%)			
MD Anderson	5 (8)	8 (13)	0.58
University of Pittsburgh	6 (10)	4 (7)	
Wake Forest University	50 (82)	48 (80)	
Pathology, *n* (%)			0.54
Low grade	40 (68)	42 (74)	
High grade	19 (32)	17 (26)

**Table 2:** Survival outcomes

Outcomes	Mitomycin C (*N* = 61)	Oxaliplatin (*N* = 60)	*p*-value
10-year survival	56.2% (7.2)	47.5% (8.4)	0.83
10-year PFS	45.2% (8.4)	50.4% (6.7)	0.95
Median survival	NR (> 5.6 years)	9.1 years	
Low grade			
5-year survival	75.1% (7.3)	82% (6.2)	0.78
10-year survival	68.3% (8.1)	62.1% (10.1)	
High grade			
5-year survival	43.8% (12.4)	10.4% (9.7)	0.12
10-year survival	NR	NR	

The patients were approximately 92% Caucasian, 7% African American, and 1% Hispanic or Asian. Patient recruitment was from Wake Forest (80% of cases), M.D. Anderson (13% of cases), and University of Pittsburgh (7% of cases). The average peritoneal carcinomatosis index scores were similar with 18.0 (SD 10.0) in the mitomycin C group versus 17.9 (SD 9.4) in the oxaliplatin group. A total of 20% percent of patients in the mitomycin C group received neoadjuvant chemotherapy, compared with 19% in the oxaliplatin arm (*p* > 0.99). The appendiceal tumor was low grade in 71% and high grade in 29%. Resection (R) scores were similar between groups, with 54% and 46% achieving R0/1 and R2 resections in the mitomycin C group versus 51% and 49%, respectively, for the oxaliplatin group.

**Fig. 2:**
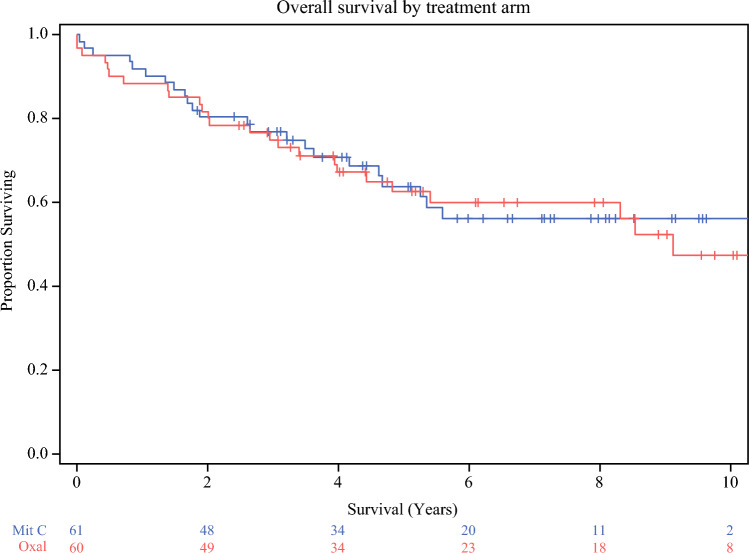
Overall survival by treatment arm

**Fig. 3:**
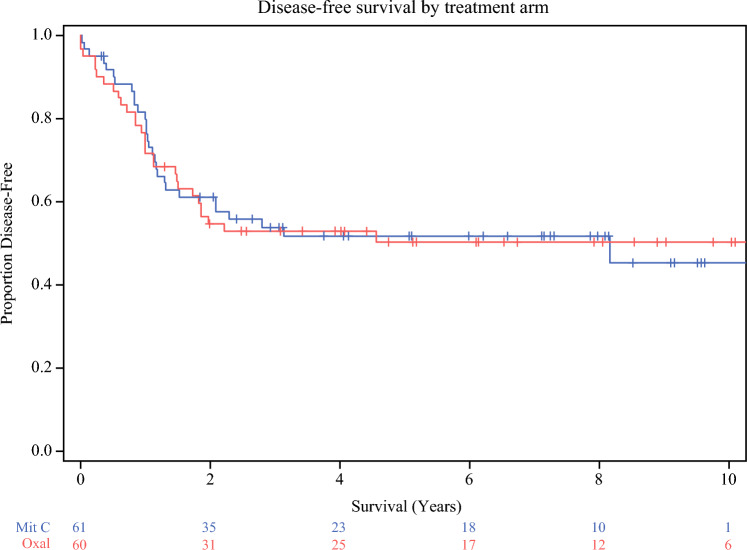
Disease-free survival by treatment arm

We looked at overall survival in both arms and stratified by grade and resection status. Our results are as follows: 10-year survival rate of the overall population was 56.2% (SE 7.2) with mitomycin C and 47.5% (SE 8.4) with oxaliplatin; *p* = 0.83 (Figs. 2 and 3). Median survival was 9.1 years after HIPEC with oxaliplatin, and median not reached for the mitomycin C group (> 5.6 years). The 10-year progression free survival rate in the mitomycin C group was 45.2% (SE 8.4) compared with 50.4% (SE 6.7) in the oxaliplatin group; *p* = 0.95.

When stratified by grade; 5- and 10-year survival for patients with low-grade disease was 78.5% (SE 4.8) and 62.5% (SE 7.3), respectively. For patients with high-grade disease, the 5-year survival rate was 29.5% (SE 9.1), with no patient attaining 10-year survival. The overall survival is significantly better for patients with low-grade disease compared with high grade, irrespective of chemotherapeutic agent; *p* < 0.001.

When comparing survival for each chemotherapeutic agent by disease grade, in patients with low-grade disease who underwent CRS/HIPEC with mitomycin C (*N* = 40), 5-year survival was 75.1% (7.3) compared with 82% (6.2) in patients with low-grade appendiceal neoplasms who had HIPEC with oxaliplatin (*N* = 41). The 10-year survival in patients with low-grade appendiceal neoplasms who underwent HIPEC with mitomycin C was 68.3% (8.1) compared with 62.1% (10.1) for the oxaliplatin cohort in this group; *p* = 0.78.

When stratified by resection status, there are no differences between HIPEC with mitomycin C and oxaliplatin. The median has been reached only in the R2 patients, so this summary will focus on estimated survival as of the last death in each resection and treatment group combination. In R0 patients, mitomycin C (*N* = 13) had 84.6% (SE 10.0) survival at 2 years (this is the last known failure) compared with 50.0% (SE 15.6) at 4.8 years (*N* = 14), with a *p*-value of 0.19. For R1, mitomycin C (*N* = 20) had 64.2% (SE 12.0) survival at 5.6 years (the last death) compared with 78.1% (SE 13.1) at 9.2 years, also the last death in the oxaliplatin arm (*N* = 23), with a *p*-value of 0.10. The mitomycin C (*N* = 28) median for R2 resection patients is 4.7 years; oxaliplatin (*N* = 23) subjects had a median survival of 4.0 years (*p* = 0.35).

In patients with high-grade appendiceal neoplasms who underwent CRS/HIPEC with mitomycin C (*N* = 16), 5-year survival was 43.8% (12.4) and 10.4% (9.7) in the oxaliplatin group (*N* = 12). For patients with high-grade disease, 10-year survival was not reached in either group. Median survival in mitomycin C group was 3.6 years and 1.9 years in oxaliplatin group; *p* = 0.12.

## Discussion

The rarity and variety of appendiceal primary lesions makes study of this disease difficult. Although the incidence has been increasing for over a decade,^1^ several issues have made completing clinical trials difficult.^23^ The paucity of available funding support and rarity of the appendiceal tumors have made randomized trials very challenging to complete. This study is the first randomized trial for cancer of the appendix with oncologic endpoints ever completed to the best of our knowledge.

Many oncologic trials follow patients for 5 years to consider long-term outcomes. However, appendiceal neoplasms can have a longer, more protracted course, making 10 years a more realistic measure of long-term outcome. With that in mind, the literature describing 10-year survival rates of patients undergoing CRS/HIPEC for appendiceal neoplasms is scarce. This study showed that most patients who undergo CRS and HIPEC for appendiceal neoplasms can experience long-term survival. This agrees with prior reports that have demonstrated that long-term survival is possible, even for high-grade disease, if complete cytoreduction is obtained.^6,9,18,19,23^

The use of oxaliplatin has been of great interest in European centers and has been a standard agent for HIPEC there. The most commonly cited dose of oxaliplatin has been 460 mg/M^2^ with a 30-min perfusion, frequently given synchronously with intravenous 5-fluorouracil and leucovorin.^24^ The 30-min perfusion has been challenged as being too short for full efficacy. Our phase 1 trial of oxaliplatin with a 120-min perfusion found a maximally tolerated dose of 200 mg/M^2^ with nearly double the area under the curve as the European dose.^12^ Retrospective analyses have reached differing conclusions on which agent is superior.^13–16^ Our previous retrospective trial, as well as a report from Holland,^16^ found no difference between mitomycin C and oxaliplatin.^13^ An Australian report suggested oxaliplatin to have a better survival for colorectal primary,^15^ and a group report from the American Society for Peritoneal Surface Malignancies suggested mitomycin C might be a better agent.^14^ Therefore, we chose to compare a standard dose of mitomycin C with a dose of oxaliplatin compatible with a longer (2 h) perfusion time.

Our population had an overall 56.2% 10-year survival rate with mitomycin C and 47.5% with oxaliplatin. In a prospective study of a retrospective national database with patients who underwent CRS and HIPEC with mitomycin C from the United Kingdom, Aziz et al.^25^ reported a 5-year survival of 55.5% and a 36% rate of disease-free survival at 5 years, with a median PCI of 6 (0–34). A study from the Netherlands^26^ looked at the difference in outcomes of 297 patients undergoing CRS/HIPEC with mitomycin C versus oxaliplatin for colorectal cancer, and showed no difference in overall survival for the groups. Median OS was 30.7 months in the mitomycin C group versus 46.6 months in the oxaliplatin group; *p* = 0.181.

The pathologic grade of appendiceal tumors is important to highlight. Our study showed similar 5- and 10-year survival rates when comparing both chemotherapeutic agents in patients with low-grade disease. On the contrary, for patients with high-grade disease, although the sample size is small and the difference does not reach significance, the estimated survival with use of mitomycin C was superior at 5 years (43.8% versus 10.4%).

Other studies have described differences in outcomes using these two chemotherapeutic agents, however, their survival outcomes are not reported.^27^ Masckauchan et al.^28^ reported a 5-year survival rate of 40.1% in their cohort of patients with appendiceal carcinoma who underwent CRS/HIPEC with oxaliplatin. Of note, in this study concomitant intravenous chemotherapy was administered with 5FU, which differs from our protocol, and no comparison with other HIPEC agents was performed.

Since the initial publication of this trial, additional data have been published comparing oxaliplatin with mitomycin C in the perfusate of HIPEC procedures. Our group has evaluated the optimal agent duration and temperature of HIPEC using an organoid platform.^29^ When utilized for 120 min at elevated temperatures, mitomycin C and oxaliplatin have similar effects on tumor cell viability in this model. A recent retrospective multi-institutional cohort study by Fisher and colleagues^30^ reported outcomes of more than 2000 patients with peritoneal disease secondary to colorectal cancer and compared their outcomes after cytoreduction and HIPEC with oxaliplatin versus mitomycin C. In their study, median OS was higher in the oxaliplatin group (47 months versus 39 months, *p* < 0.001); with a 5-year survival of 42% with oxaliplatin and 33.6% in the mitomycin C group (*p* < 0.001). We should note that their population is neither randomized nor exactly comparable to ours, given the colorectal primary in their study. In addition, the median PCI in this population was lower (8 for both groups) with > 90% complete cytoreduction. Approximately 30% of the patients perfused with oxaliplatin had irinotecan added to the perfusate, and were perfused for 30 min, compared with a 90-min perfusion in their mitomycin C group; which differs from our protocol as well, which precludes drawing definitive conclusions.

Even though our initial report found differences in hematologic toxicities with both agents, in the long term, survival is not affected by these short-term toxicities. Similar results have been reported in the colorectal and GYN oncology population.^13,31^ The data also confirm that for low-grade completely resected (R0/1/R2a) disease, systemic chemotherapy is unlikely to improve long-term survival. Given the low rates of response and the excellent outcomes with cytoreductive surgery and HIPEC, we see a very limited role for systemic treatment in this setting. Additionally, a retrospective review of 134 cases of repeat CRS/HIPEC for low-grade appendiceal neoplasms at our institution^32^ showed a 16.9% conversion from low-grade to high-grade disease in patients who received systemic chemotherapy, which significantly correlated with survival after repeat CRS/HIPEC. High-grade disease, on the contrary, had much poorer outcomes, and such cases should be considered for systemic preoperative and/or postoperative chemotherapy.^33–35^

Although it has previously been attempted for appendiceal cancer, no trial has compared aggressive cytoreduction with and without HIPEC in this population. The findings of the PRODIGE 7 trial^24,36^ are not applicable in this setting since the appendiceal lesions are remarkably different from colonic adenocarcinoma on genomic analysis,^37,38^ despite the appendix being anatomically part of the colon. Further, retrospective studies with and without intraperitoneal adjuvants have shown advantages for intraperitoneal adjuvants.^39^

The Peritoneal Surface Oncology Group International (PSOGI) performed a retrospective review of their registry including 1924 patients with peritoneal disease secondary to appendiceal mucinous neoplasms treated with or without HIPEC in a 24-year period.^40^ Their study showed that the use of HIPEC during cytoreduction was associated with better overall survival.

The current study represents a sizeable cohort of a very rare disease and is the first completed randomized trial of its kind, however, it has several limitations that must be considered. The selection of sites for this study was limited to institutions that have been through the lengthy learning curve, and results at less experienced centers may not be as good.^41^ This study was not powered sufficiently to be able to define small differences in survival, and a much larger trial might be able to define a survival difference, if such a difference exists.

In conclusion, oxaliplatin and mitomycin C have similar long-term efficacy for HIPEC in patients with appendiceal neoplasms and peritoneal dissemination. Long-term survival is experienced by most patients after CRS and HIPEC for appendiceal neoplasms. In high-grade disease, mitomycin C may have superior survival outcomes. Short-term differences in hematologic toxicity do not affect long-term outcomes. Recurrence is less likely after 5 years of stability. We suggest that future studies plan for long-term (10-year) follow-up of participants after interventions**.**
